# Synergistic Photothermal-Chemotherapy Based on the Use of Biomimetic Magnetic Nanoparticles

**DOI:** 10.3390/pharmaceutics13050625

**Published:** 2021-04-28

**Authors:** Ylenia Jabalera, Alberto Sola-Leyva, María P. Carrasco-Jiménez, Guillermo R. Iglesias, Concepcion Jimenez-Lopez

**Affiliations:** 1 Department of Microbiology, Faculty of Sciences, University of Granada, 18071 Granada, Spain; yjabalera@ugr.es (Y.J.); cjl@ugr.es (C.J.-L.); 2 Department of Biochemistry and Molecular Biology I, Faculty of Sciences, University of Granada, 18071 Granada, Spain; albertosola@ugr.es; 3 Department of Applied Physic, Faculty of Sciences, University of Granada, 18071 Granada, Spain

**Keywords:** biomimetic magnetic nanoparticles, combined tumor therapy, doxorubicine, HepG2 cancer cell line, MamC, magnetite nanoparticles, photothermia

## Abstract

MamC-mediated biomimetic magnetic nanoparticles (BMNPs) have emerged as one of the most promising nanomaterials due to their magnetic features (superparamagnetic character and large magnetic moment per particle), their novel surface properties determined by MamC, their biocompatibility and their ability as magnetic hyperthermia agents. However, the current clinical application of magnetic hyperthermia is limited due to the fact that, in order to be able to reach an effective temperature at the target site, relatively high nanoparticle concentration, as well as high magnetic field strength and/or AC frequency are needed. In the present study, the potential of BMNPs to increase the temperature upon irradiation of a laser beam in the near infrared, at a wavelength at which tissues become partially transparent, is explored. Moreover, our results also demonstrate the synergy between photothermia and chemotherapy in terms of drug release and cytotoxicity, by using BMNPs functionalized with doxorubicin, and the effectiveness of this combination therapy against tumor cells in in vitro experiments. Therefore, the findings of the present study open the possibility of a novel, alternative approach to fight localized tumors.

## 1. Introduction

Nanomedicine has emerged in recent decades as the alternative to solve the limitations of current therapeutic tools in the treatment of localized diseases, especially cancer. Several smart nanomaterials have been developed with the goal of using them both as nanocarriers for a targeted chemotherapy, and as agents able to exert a physical effect on the target tissues [1,2]. In this context, one of the most widely studied physical effects is magnetic hyperthermia, or local heating induced by the application of alternating magnetic fields. Magnetic hyperthermia results in the production of localized heat with temperatures raising up to 42–46 °C at the tumor site, which is able to kill cancer cells through apoptosis or necrosis [3,4,5]. Moreover, it has been demonstrated to trigger the release of the relevant molecule from the nanocarrier [6,7,8,9,10], thus increasing the efficacy of the treatment by the synergy of chemotherapy–magnetic hyperthermia, which pose an advantage compared to other nanoformulations in which the drug release at the target is the limiting step [11]. The localized heat may also be used to pre-treat the tumor to optimize the efficiency of further treatments, since it increases vascular leakage and promotes the accumulation of the nanoparticles [12]. However, the current clinical application of magnetic hyperthermia is still under development [12], somehow limited due to the need of a relatively high nanoparticle concentration, as well as moderately high magnetic field strengths and/or AC frequencies to reach the effective temperature at the target site [13,14,15,16].

Therefore, new alternatives need to be raised to be able to combine chemo-physical strategies to potentially increase the function of nanomedicine [17]. In this context, a relatively novel physical mechanism also able to induce hyperthermia is so-called photothermal therapy, in which, upon irradiation with a laser, the agent absorbs the electromagnetic energy and converts it into heat. In addition to producing cell damage associated with heat, photothermia may also trigger drug release from the nanoassembly, just as hyperthermia does. In this strategy, the use of nanoparticles with sufficient absorbance in the near infrared (NIR) biological window (650–900 nm), where the tissues become partially transparent without light absorption, results in both high light responsiveness at low laser powers, and also in-depth penetration with low concentration of the photothermal agent [18,19,20]. Until now, the named photothermal agents have been referred to as carbon nanotubes or gold nanoparticles (plasmonic compounds) which, although displaying an exceptional optical absorbance in the NIR window, also present some drawbacks that limit their clinical applications, such as difficulties in guiding and concentrating them at the target site by external means, persistency and low biodegradability [20,21,22]. 

Therefore, in order to exploit the potential of photothermal therapy in clinics, finding a good photothermal agent becomes crucial. This agent should comply with the following requirements: being biocompatible, biodegradable, able to absorb NIR radiation with an efficient light-to-heat conversion and, ideally, prone to external guidance/concentration to the target site, thus allowing the combination of photothermal therapy with other directed therapies, for instance, chemotherapy [23]. 

In this context, magnetic nanoparticles have already proven their potential in biomedical applications, mainly due to their ability to be externally controlled by means of a magnetic field, but also because of their biocompatibility, biodegradability and ease of synthesis [24]. In fact, the Food and Drug Administration agency (FDA) has approved their use in clinics as magnetic resonance imaging (MRI) contrast agents, and hence they would be candidates as magnetic hyperthermia agents (MagForce AG, Germany) [25,26]. In addition, iron oxide nanoparticles exhibit an extended optical absorbance, which decreases from the near UV to the NIR, thanks to the electronic transition between Fe^2+^ and Fe^3+^. In fact, recently, iron oxide nanoparticles have been tested for photothermal therapy in vitro and in vivo [13,14,22,27,28,29,30]. Therefore, they offer great possibilities to be considered for cancer therapy as a drug delivery system, magnetic hyperthermia and photothermal agent. 

Among iron oxide nanoparticles, MamC-mediated (MamC: magnetosome membrane-associated protein from *Magnetococcus marinus* MC-1) biomimetic magnetic nanoparticles (BMNPs), synthetized inorganically in the presence of the mentioned protein, have emerged both as a good nanocarrier and magnetic hyperthermia agent, allowing the efficient combination of both therapies in the context of cancer. This is due to the novel surface properties of BMNPs conferred by MamC, which compared to traditional inorganic magnetic nanoparticles, ease the functionalization of the nanoparticle with the relevant molecule by means of electrostatic interaction, and allow its release under acidic conditions, which naturally occur at the tumor site. Moreover, their larger size (~40 nm) added to the fact that, in contrast with the inorganic ones, BMNPs do not need to be coated prior to functionalization, make these biomimetic nanoparticles exhibit fairly good magnetic susceptibility upon the application of an external magnetic field, while maintaining their superparamagnetic character. Moreover, BMNPs have proven cytocompatible, non-toxic, biodegradable and easy to produce by an eco-friendly, cost effective and scalable method [31,32,33,34]. All of these characteristics create an advantage over traditional inorganic magnetic nanoparticles and put BMNPs in focus as a novel potential agent to combine different therapies against targetable and/or localized diseases.

Here, for the first time, the potential of BMNPs to act as photothermal agents, and their predicted effectiveness in combining directed chemotherapy and photothermal therapy in the context of cancer, are explored. For this purpose, BMNPs were functionalized with the model chemotherapy agent doxorubicin (DOXO) to form DOXO-BMNP nanoassemblies, and their ability to respond to a NIR laser was analyzed. Finally, the internalization of the nanoassembly, and the in vitro antitumor activity of the dual treatment by using DOXO-BMNPs, were investigated using HepG2 human hepatoma cell lines as a cell model. 

## 2. Materials and Methods

### 2.1. BMNP Production

MamC was expressed and purified as recombinant protein following the protocol described in [31]. Briefly, *Escherichia coli* TOP10 (Life Technologies: Invitrogen, Grand Island, NY, USA) was transformed with the plasmid pTrcHis-TOPO (Life Technologies: Invitrogen) used as a vector of the MamC protein-coding gene (Mmc1_2265) coupled to a hexahistidine tag coding sequence at its 5′ terminus. The cells were grown at 37 °C in Luria-Bertani (LB) broth supplemented with ampicillin. After 5 h of incubation with isopropyl-β-d-thiogalactopyranoside (IPTG, Fisher BioReagents, Pittsburgh, PA, USA), the expression of the recombinant MamC was induced. Once expressed, the purification of the protein was carried out under denaturing conditions by fast protein liquid chromatography (FPLC, GE Healthcare), using immobilized metal affinity chromatography (IMAC, GE Healthcare, Chicago, IL, USA). Finally, fractions containing MamC were refolded at 4 °C through dialysis by using 1 L of the buffers A (Tris 50 mM, NaCl 150 mM, urea 6 M, pH 8.5) and B (Tris 50 mM, NaCl 150 mM, pH 8.5) as the starting and end points, respectively. The purity of the protein was evaluated by SDS-PAGE electrophoresis.

The synthesis of BMNPs was carried out at 1 atm total pressure and 25 °C from oxygen-free solutions containing 3.5 mM Na_2_CO_3_, 3.5 mM NaHCO_3_, 2.78 mM Fe(ClO_4_)_2_, 5.56 mM FeCl_3_ and 10 μg mL^−1^ recombinant MamC, at a pH value of 9 (protocol described in [31,35]). All experiments were performed under anaerobic conditions inside an anaerobic Coy chamber (96% N_2_/4% H_2_, Coy Laboratory Products, Grass Lake, MI, USA). Samples were incubated for 30 days and then the solids were magnetically concentrated and washed with deoxygenated Milli-Q water. Finally, the solid precipitated was stored in HEPES buffer (HEPES 10 mM, NaCl 150 mM, pH 7.4) inside the Coy Chamber at 25 °C.

### 2.2. BMNP Functionalization

Biomimetic magnetic nanoparticles (BMNPs) were functionalized with the chemotherapeutic drug doxorubicin (Sigma-Aldrich, Madrid, Spain), following the protocol described in [32]. Briefly, 5 mg of BMNPs were mixed with 1 mg mL^−1^ DOXO suspended in HEPES buffer at pH 7.4 for 24 h, inside hermetically closed bottles to avoid magnetite oxidation at 25 °C, in a rotating wheel. At the end of each incubation, nanoassemblies were collected with a magnet and washed three times with HEPES buffer. Supernatants and the washings were mixed and the amounts of DOXO measured by UV–vis spectroscopy (λ = 490 nm). This measured unbound DOXO was detracted from the amount initially incubated, so the amount of bound DOXO could be indirectly determined and, from that, the efficiency of the functionalization was assessed. 

### 2.3. Nanoparticle and Nanoassembly Characterization

Transmission electron microscopy (TEM) analyses were performed with a STEM Philips Model CM20 microscope on ultrathin sections (50–70 nm) prepared by embedding the nanoparticles in Embed 812 resin and then cutting them using a Reichert Ultracut S microtome (Leica Microsystems GmbH, Wetzlar, Germany). ImageJ 1.47 software was used to measure particle sizes on multiple micrographs with more than 1000 nanoparticles measured to ensure reproducibility. The hydrodynamic particle size of the samples at pH 7.4 was measured by dynamic light scattering (DLS) using a Nano-ZS instrument (Malvern Instruments, Worcestershire, UK). Electrophoretic mobility measurements were carried out in a Zetameter Nano-ZS (Malvern Instruments, Malvern, UK) at 25 °C. Stock suspensions of BMNPs were prepared in 20 mL of oxygen-free NaClO_4_ (10 mM). Aliquots of 200 µL from each stock were suspended in flasks containing oxygen-free NaClO_4_ and the pH was adjusted from 2 to 9. Samples were sonicated for 2 min, and the electrophoretic mobility was immediately measured. All measurements, done in triplicate for each sample, were carried out at 25 °C using disposable plastic cuvettes. 

Optical spectra of both BMNPs and DOXO-BMNPs nanoassemblies were obtained by using a UV–vis spectrometer, the Jenway™ 6705 (Jenway, Staffordshire, United Kingdom), in the 200–900 nm spectral range.

Fourier-transform infrared (FTIR) analysis was carried out by using a FTIR spectrometer (model 6600, Jasco, Japan) equipped with an attenuated total reflection (ATR) diamond crystal window (ATR ProOne). The surface of the sample was pressed against the ATR window and infrared spectra were acquired. A total of 100 scans were collected in the wavenumber range from 4000 to 400 cm^−1^, at 2 cm^−1^ resolution.

### 2.4. Photothermal Measurement in Aqueous Suspension 

Experiments were run in Eppendorf tubes (0.5 mL) containing 0.2 mL of suspension of nanoparticles in HEPES buffer. Nanoparticle concentrations were adjusted to [Fe] = 19 mM. During the experiments, each sample was irradiated from the top with a NIR laser (λ = 808 nm) at 0.5, 1 and 2 W cm^−2^, and visualized with a thermographic camera (Flir 60 with 320 × 240 pixels, IR resolution and thermal sensitivity <0.045 °C; FLIR Systems, Inc., Wilsonville, OR, USA), in order to measure temperature variations. Temperature measurements were taken at the top, middle and bottom of the suspension. 

### 2.5. Effect of Photothermia on Nanoassembly Stability and Drug Release 

The stability of the DOXO-BMNP nanoassemblies suspended in HEPES buffer at pH 7.4 was studied with and without irradiation. Identically, the release of DOXO from BMNPs was studied on suspensions of DOXO-BMNPs in citrate buffer at pH 5, before and after irradiation. During the experiments, the NIR laser (λ = 808 nm) at 2 W cm^−2^ was applied to the suspension of DOXO-BMNPs ([Fe] = 19 mM, 0.2 mL) for 40 min either in HEPES or in citrate buffer, and DOXO release was determined at different time intervals. The control experiments were run under identical conditions, but with no irradiation applied. At specific intervals, the nanoassemblies were magnetically separated from the supernatant and they were resuspended in fresh buffer to continue the remaining time intervals (every 5 min). Finally, all supernatants were measured by UV–vis spectroscopy at λ = 490 nm and the concentration of DOXO released from the nanoassembly was determined. Each experiment was performed in triplicate for all conditions. 

### 2.6. Cell Culture 

The HepG2 cell line, from human hepatoblastoma, was supplied by the European Collection of Animal Cell Cultures (Salisbury, UK). This cell line uses Minimum Essential Medium (MEM) enriched with 10% heat-inactivated fetal bovine serum (FBS) and 2 mM L-glutamine, and supplemented with 100 U mL^−1^ penicillin and 100 μg mL^−1^ streptomycin, at 37 °C in a 5% CO_2_ humidified atmosphere. Cell subcultures were carried out when reaching cell confluence or by experimental requirements.

### 2.7. Internalization of BMNPs

In order to evaluate the amount of BMNPs internalized, HepG2 cells were seeded in 12-well plates (300,000 cells per well) and treated with BMNPs 300 ug mL^−1^ ([Fe] = 3.8 mM) for 24 h. The non-internalized BMNPs were washed away with PBS, afterwards cells were trypsinized and transferred to 2 mL tubes. In the final step the particles were centrifuged at 1000 rpm for 5 min. Then, in order to dissolve the cell pellet, 37% HCl/10% H_2_O_2_ was added and maintained for 20 min at room temperature. One milliliter of 1% potassium thiocyanate in Milli-Q water was immediately added to the tubing and the absorbance at 490 nm was measured. A standard calibration curve was used to obtain the endogenous iron of the cells.

### 2.8. In Vitro Photothermal and Cytotoxicity Assays

HepG2 cells were seeded onto 12 well-plates at 300,000 cells per mL and three different treatments were applied individually (over 24 h): (1) BMNPs (300 ug mL^−1^, [Fe] = 3.8 mM), (2) DOXO-BMNP nanoassemblies (300 ug mL^−1^ [Fe] = 3.8 mM), (3) DOXO or (4) just medium MEM/10% FBS, this experiment acting as control. After the 24 h treatment, cells were tripsinized and resuspended in 200 μL of fresh medium MEM/10% FBS, and then transferred to Eppendorf tubes (0.5 mL), obtaining a final cell concentration of 1.5 million cell/mL. Thus, the nanoparticle concentration increased up to 1.5 mg mL^−1^ ([Fe] = 19 mM). Then, each sample was irradiated for 600 s from the top with the NIR laser set at 2 W cm^−2^. In parallel, the same experimental conditions were tested without exposing the cells to the laser. After exposure, 200 μL of each sample was transferred to each well of a 96-well black plates and 20 μL of resarzurin 1 mM in PBS was added (R7017, Sigma-Aldrich, Spain). The reaction was incubated in the dark for 2 h and the fluorescence intensity was measured at λ_ex_ = 535/λ_em_ = 590 nm.

### 2.9. Statistical Analysis

Statistical analyses were performed using GraphPad Prism version 8.4.2 for Windows, GraphPad Software (GraphPad Prism, San Diego, CA, USA). For in vitro biological analysis, data represent means ± SEM (standard error of mean) of three independent experiments performed in triplicate, and statistical analyses were carried out using two-way ANOVA, with a Bonferroni’s post-test for grouped analysis. Statistical differences between the treatments were considered significant when *p*-values were *p* ≤ 0.05 (*), *p* ≤ 0.01 (**) and *p* ≤ 0.001 (***).

## 3. Results and Discussion

### 3.1. BMNP and Nanoassembly Characterization

**Figure 1:**
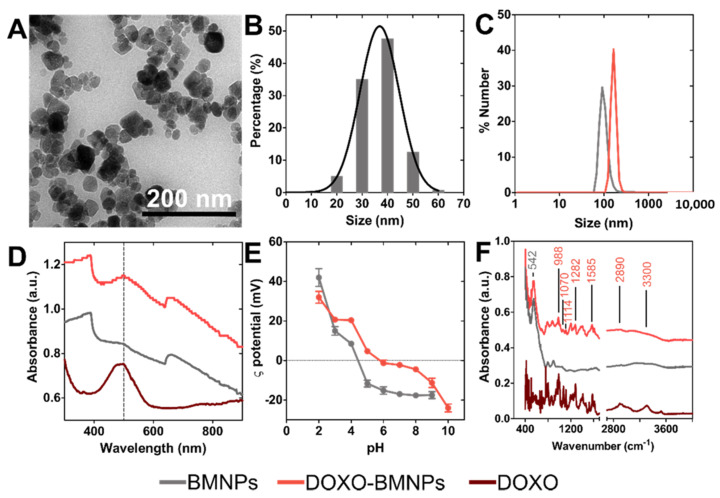
Nanoparticle and nanoassembly characterization. (**A**) Transmission electron microscopy (TEM) images of biomimetic magnetic nanoparticles (BMNPs). (**B**) BMNPs’ size distribution measured by TEM. (**C**) BMNPs’ and DOXO-BMNPs’ size distribution measured with DLS (number distribution). (**D**) UV−vis−NIR absorbance spectra, (**E**) ζ-potential and (**F**) ATR-FTIR spectra of DOXO, BMNP and DOXO-BMNP nanoassemblies. In panels (**C**–**F**), the gray (black) color corresponds to BMNPs (DOXO-BMNP nanoassemblies).

The BMNPs crystals obtained display well defined faces with different morphologies including square, rectangular or rhombic geometries (Figure 1A). The average magnetic core size, measured by TEM, was equal to 39 ± 7 nm, but crystal sizes could be found within the interval 10 to 70 nm. Measurements of the hydrodynamic diameter, carried out by DLS, yielded sizes of BMNP and DOXO-BMNP nanoassemblies of 100 ± 20 and 160 ± 30 nm, respectively (Figure 1B,C). These figures indicate a fairly good colloidal stability, since the aggregates were composed of a maximum of 3–4 BMNPs. The UV−vis−NIR absorbance spectra of BMNPs and DOXO-BMNPs (Figure 1D) showed the ability of these biomimetic nanoparticles and nanoassemblies to exhibit a high absorbance in the NIR region. This is due to the electronic transitions between Fe^2+^ and Fe^3+^ ions that form magnetite, which is the mineral phase of the BMNPs. These data are in good agreement with those reported for other synthetic magnetic nanoparticles [18,36]. 

The ζ potential values for BMNPs revealed that these nanoparticles were negatively charged (−19 mV) at physiological pH (Figure 1E). This feature allows electrostatic repulsion among nanoparticles and that, along with their superparamagnetic behavior [37], prevents the aggregation of the magnetic crystals in the absence of an external magnetic field and, therefore, ensures relatively good colloidal stability, as our data show (Figure 1B,C). On other hand, the fact that BMNPs were negatively charged at pH 7.4 allowed their functionalization with the relevant molecule based on electrostatic interactions. Most of the functional groups at the BMNP surface which are responsible for the negative charge at physiological pH are those of MamC, which show a high percentage (26.3%) of acidic and polar amino acids on its structure [31,33,38]. This poses an advantage over other magnetic nanoparticles in terms of functionalization, as many of the synthetic inorganic magnetic nanoparticles are nearly uncharged at physiological pH values and require further coating in order to be functionalized [24,39]. In this context, at the pH value at which functionalization is performed (7.4), BMNPs are negatively charged while DOXO molecules are positively charged in aqueous solution, due to the –NH_3_ groups present in their structure, thus allowing the coupling based on electrostatic interaction [32]. 

The occurrence of this functionalization agrees with the ζ potential values of DOXO-BMNP nanoassemblies (Figure 1E), which showed values of −3 mV at physiological pH. This new value indicates that the negatively charged groups of MamC previously present on BMNPs were now blocked, probably by the binding to DOXO molecules. This coupling was further confirmed by UV−vis absorbance measurements (Figure 1D) and ATR-FTIR spectra (Figure 1F). Absorbance measurements showed that, compared to BMNPs, DOXO-BMNPs displayed a new peak at 490 nm that corresponded with the absorbance of DOXO (Figure 1D). In addition, ATR-FTIR data showed adsorption bands that were different in BMNPs and DOXO-BMNPs, which further confirmed the coupling. The peak at 542 cm^−1^, characteristic of Fe-O bond in magnetite, is presented in both spectra [40]. However, the ATR-FTIR spectra of DOXO-BMNPs showed additional peaks at 3300, 2890, 1585, 1282, 1114, 1070 and 988 cm^−1^ that are characteristic of the DOXO molecule [41] (Figure 1F). 

### 3.2. Photothermal Response

**Figure 2:**
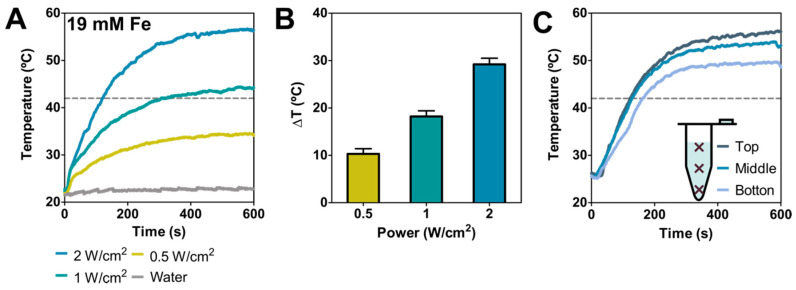
Nanoparticles’ photothermal response in suspension. (**A**) Temperature (measured at the top of the suspension) as a function of irradiation time at 0.5, 1 and 2 W cm^−2^ ([Fe] = 19 mM and water as a control). (**B**) Temperature increases for different laser powers after 5 min of treatment. (**C**) Temperature as a function of irradiation time at 2 W cm^−2^ ([Fe] = 19 mM) recorded at different distances from the irradiation point, being the “top” position.

BMNPs photothermal response was evaluated in aqueous solutions by using, as mentioned, a laser with power ranging from 0.5 to 2 W cm^−2^ (values that avoid collateral tissue damage in vivo [18]) and using an Fe concentration of 19 mM (concentration at which photothermal effect is not saturated [13,18,30]), that corresponds to 1.5 mg mL^−1^ of BMNPs. Figure 2A shows the temperature increase curves obtained at different laser powers, showing an efficient and relevant temperature increase (>10 °C) under all conditions tested. Specifically, the average final temperature rise obtained after 10 min was 10.3 ± 1.1, 18.2 ± 1.2, 29.2 ± 1.1 °C at 0.5, 1 and 2 W cm^−2^, respectively (Figure 2B), the fastest increase occurring within the first 200 s of the irradiation (Figure 2A). 

To evaluate sample homogeneity in terms of temperature increase, the temperature at three different positions within the samples was recorded. The data from these experiments show that the temperature rise varied slightly along the tubing (Figure 2C). In fact, the absolute final temperature varied within ~6.4 °C from the top to the bottom, resulting in a temperature decrease of ~0.053 °C mm^−1^ as the distance increased from the irradiation point.

The average final temperature increments obtained in this study, when the BMNPs were used as photothermia agents, were higher than those obtained when the same BMNPs were used as magnetic hyperthermia agents, at field intensities and frequencies within the range allowed for therapeutics (temperature increment values ranging from 7.8 to 10.2 °C, using an Fe concentration of 130 mM) [37,42]. It is interesting to note that, at BMNP concentrations 10 times lower, laser irradiation (under conditions reported safe for clinical applications) induces a thermal response in BMNPs that is twice or three times stronger than that induced by an alternating magnetic field after comparable treatment times. These results open up the unexplored potential of BMNPs as photothermal mediators. 

### 3.3. Nanoformulation as DOXO Nanocarrier

**Figure 3:**
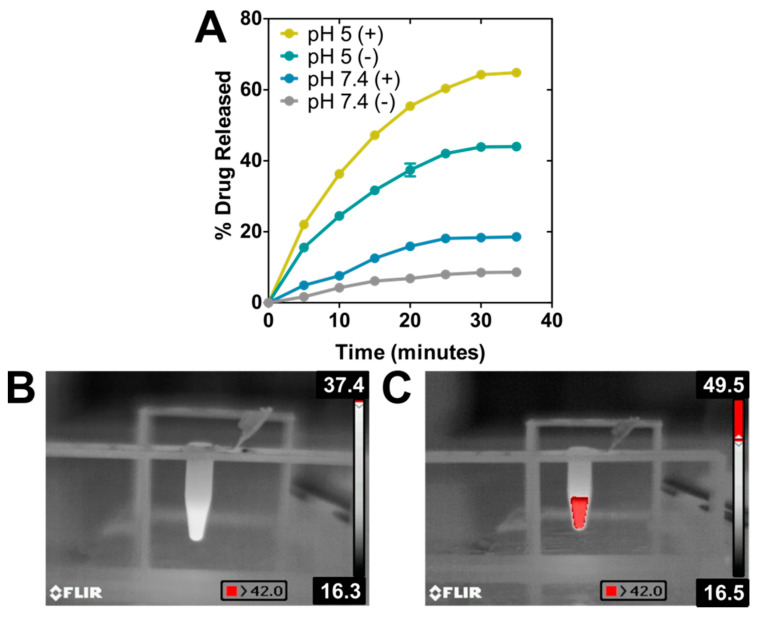
BMNPs as drug nanocarriers. (**A**) DOXO release temporal curves under the presence (+) or absence (−) of NIR-laser power of 2 W cm^−2^ at physiological and acidic pH ([Fe] = 19 mM). Thermographic photographs of samples without (**B**) and with (**C**) BMNPs irradiated with the laser.

The ability of the nanoassembly to perform as a drug nanocarrier was evaluated at pH 7.4 (physiological pH) and pH 5 (mimicking the environment in the endosomal/lysosomal compartment [43]), both with and without photothermia (Figure 3A, Table 1). During the first 30 min of the experiment, the DOXO release from the nanoassembly at physiological pH was very low, with release efficiency (DR) values that did not exceed 8% of the adsorbed drug, thus indicating the stability of the nanoformulation at physiological pH values, as it has been previously proven by other studies [32,40]. However, performing this experiment under sample irradiation, DR values increased up to 20% within the same time interval. The increase of temperature reached upon photothermia probably provided enough thermal energy to weaken the electrostatic bond that was keeping DOXO attached to BMNPs, thus accelerating the kinetics of DOXO release. These results are different than those occurring following magnetic hyperthermia with the same particles, where no DR increase was observed [40], but they are in accordance with that discussed above: the stronger effect of photothermia on BMNPs compared to that of magnetic hyperthermia. 

As expected from our previous studies, at acidic pH, ~44% of the DOXO was released from the nanoassembly within the first 30 min. As discussed, the binding of DOXO to BMNPs is mediated by electrostatic interactions and, therefore, the surface charge of the BMNPs plays a crucial role. While BMNPs are charged at physiological pH, as the environmental pH approaches the isoelectric point of the BMNPs (pH 4.3), the BMNPs become uncharged, thus releasing DOXO. Interestingly, when photothermia is applied in combination with acidic pH, DOXO DR reaches a value of ~65%. This enhanced DOXO release is the result of the synergy created by the neutralization of the BMNPs’ surface charge occurring at acidic pH values, and the increase in the BMNPs’ thermal energy induced by photothermia. In fact, pictures taken with the infrared camera (Figure 3B,C) show an evident increase in macroscopic temperature, exceeding 42.0 °C in the case of the BMNPs’ suspension. This synergy was previously shown to occur when magnetic hyperthermia treatment was applied to BMNP nanoassemblies at acidic pH values [7,40]. This is relevant, since the fact that the nanoassembly responds to active/external (photothermia) and passive/endogenous (acidic pH) stimuli would allow implementing a drug delivery strategy for spatial−temporal controlled release [1]. 

### 3.4. BMNP Internalization by Cells, and Cytotoxicity Assay In Vitro

**Figure 4:**
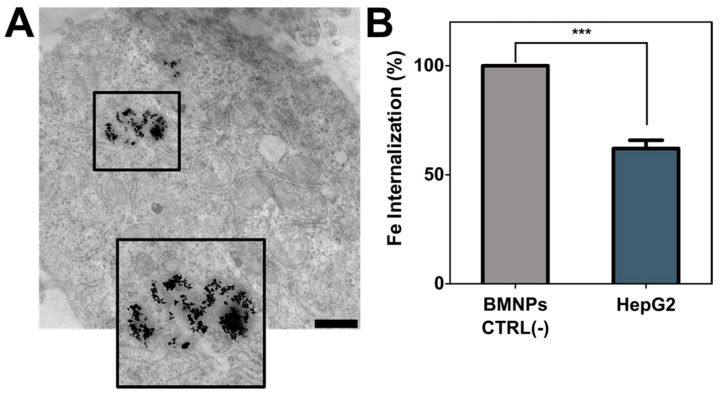
Nanoparticles’ internalization. (**A**) HepG2 cell micrography by TEM with BMNPs endocitated. Scale bar corresponds to 1 µm. (**B**) Analysis of quantity of BMNPs uptaken by HepG2 cells (BMNPs CTRL corresponds to the control without cells). These experiments were conducted twice in triplicates. *p* ≤ 0.001 (***).

As previously demonstrated, HepG2 cells internalize BMNPs by endocytosis [44] (Figure 4A). In the present study, we determined that almost 60% of BMNPs were internalized after 24 h of incubation with the nanoparticles (Figure 4B).

**Figure 5:**
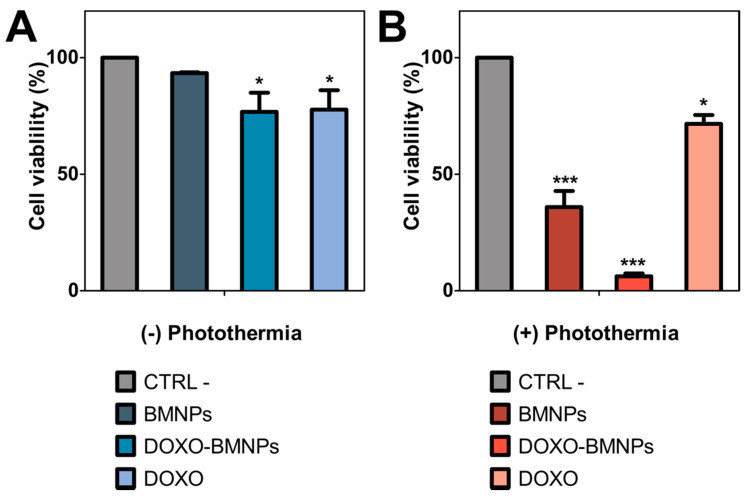
HepG2 cell viability with and without photothermia treatment in vitro. Cell viability was assessed with the resarzurin assay without (**A**) and after photothermia treatment with NIR-laser power of 2 W cm^−2^ for 600 s (**B**). *p* ≤ 0.05 (*) and *p* ≤ 0.001 (***).

As shown in Figure 5A, the cytocompatibility of BMNPs was again demonstrated, as was their cytotoxicity when loaded with DOXO [7]. In the absence of laser exposure, both soluble DOXO and DOXO-BMNP nanoassemblies produced a decrease in cell viability of up to 20%. However, when photothermia was in play, both BMNPs and DOXO-BMNPs significantly reduced cell viability. In fact, this decrease was 3 and 5 times larger in BMNPs and BMNPs-DOXO respectively, compared to that which occurred in the absence of photothermia (Figure 5B). Particularly relevant is the cytotoxic effect of the nanoassembly DOXO-BMNPs under these conditions, which roughly killed 90% of treated HepG2 cells.

These experimental results are clearly linked to the temperature increase triggered by the laser irradiation of DOXO-BMNP nanoassemblies, which also further allows a greater DOXO release from them. These results are the first evidence of HepG2 cell death following the temperature increase generated by BMNPs after exposure to laser irradiation. HepG2 cell damage caused by temperature has been already reported by other authors, as a result of a decrease in their metabolic activity [45,46] or by the damage of cell structures, which finally causes senescence and apoptosis [47]. Therefore, the results from the present study offer a potential therapy able to efficiently cause cell death based on temperature increases at low BMNP doses.

## 4. Conclusions

Photothermal therapy has emerged as a good alternative to solve the limitations of magnetic hyperthermia treatment in relation with the need of high nanoparticle concentration to induce localized hyperthermia. This study describes and characterizes a nanoassembly formed by novel biomimetic magnetic nanoparticles (BMNPs), mediated by MamC from *Magnetococcus marinus* MC-1, that serves as a DOXO nanocarrier and photothermia agent. The use of this nanoassembly allows a dual treatment thanks to the combination of directed chemotherapy and photothermia, in which the drug release and the localized heating can be remotely activated. These nanoassemblies were activated with a near-infrared laser, reaching an efficient heat conversion at low concentration of BMNPs and acceptable laser power doses. Moreover, DOXO release from the BMNPs, mainly triggered by the environmental acidic pH values, was enhanced by photothermia, due to the increase of thermal energy caused by the heating of the BMNP surface. This synergistic effect may be of use to remotely increase drug release in those nanoformulations in which this issue is the limiting step. The increased cytotoxicity resulting from the combination of both therapies was confirmed by in vitro experiments. In fact, the dual chemotherapy−photothermal treatment resulted in 90% HepG2 cell death in vitro after 600 s of treatment. Therefore, our results open the door to a combined treatment, in which chemotherapy and photothermia synergistically increase the effectiveness of the treatment, reducing BMNP doses, the intensity and the time of treatment, and therefore, the potential side effects that might be associated.

## Figures and Tables

**Table 1:** Summary of values of drug encapsulation and drug release efficiency under different conditions.

System	Drug Encapsulation Efficiency (%)	Drug Release Efficiency (DR) (%) ^a^			
		pH 7.4	pH 5	pH 7.4 + PTH ^b^	pH 5 + PTH ^b^
DOXO-BMNPs	70 ± 10	8.6 ± 0.9	44.0 ± 0.4	18.6 ± 0.4	65 ± 1

^a^ Values calculated with the equation described in [32] after 40 min of treatment. ^b^ PTH = photothermal treatment with an irradiation of 2 W cm^−2^.

## Data Availability

Not applicable.
